# Correction to “Impact of CYP2D6 Genotype and Inhibitor Use on Risperidone Metabolism in Children: Functional Insights Into the *17 and *29 Alleles”

**DOI:** 10.1111/cts.70641

**Published:** 2026-06-10

M. H. Bennett, E. A. Poweleit, S. E.Vaughn, et al., “Impact of CYP2D6 Genotype and Inhibitor Use on Risperidone Metabolism in Children: Functional Insights Into the *17 and *29 Alleles,” Clinical and Translational Science 19, no. 3 (2026): e70525, https://doi.org/10.1111/cts.70525.

In the article cited above, the listed authors in reference 25 are incorrect. The corrected reference is shown below.

25. Kehinde, O., Vaughn, S.E., Amaeze, O., Toren, P., Retke, B., Oni‐Orisan, A., and Ramsey, L.B. (2025), Cytochrome P450 2D6 *17 and *29 Allele Activity for Risperidone Metabolism: Advancing Precision Medicine Health Equity. Clin Pharmacol Ther, 118: 1152–1160. https://doi.org/10.1002/cpt.70012.

We apologize for this error.

